# Two sides of the Myc-induced DNA damage response: from tumor suppression to tumor maintenance

**DOI:** 10.1186/1747-1028-7-6

**Published:** 2012-02-28

**Authors:** Stefano Campaner, Bruno Amati

**Affiliations:** 1 Center for Genomic Science of IIT@SEMM, Istituto Italiano di Tecnologia (IIT), Via Adamello 16, 20139 Milan, Italy; 2 Department of Experimental Oncology, European Institute of Oncology (IEO), at the IFOM-IEO Campus, Via Adamello 16, 20139 Milan, Italy

**Keywords:** Myc, Replication stress, ATR, CHK1, DNA damage, DNA damage response, Tumor suppression, Cell cycle

## Abstract

Activation of oncogenes is generally associated with the induction of DNA damage response (DDR) signaling, which acts as a barrier to tumor progression. In this review we will present an overview of the DDR associated with oncogenic activation of Myc, with special focus on two opposite and paradoxical aspects of this response: (1) the role of the Myc-induced DDR in tumor suppression; (2) its role in dampening Myc-induced replication stress, thereby protecting the viability of prospective cancer cells. These opposing effects on cancer progression are controlled by two different branches of DDR signaling, respectively ATM/CHK2 and ATR/CHK1. Indeed, while ATM activity constitutes a barrier to malignant transformation, full activation of ATR and CHK1 is essential for tumor maintenance, providing important opportunities for therapeutic intervention. Thus, the Myc-induced DDR acts as a double-edged sword in tumor progression.

## Review

### c-MYC

c-MYC (henceforth MYC) is an immediate-early serum response gene essential for embryonic development, cellular proliferation and survival, and a cellular proto-oncogene that is frequently up-regulated in cancer. The Myc protein is a basic Helix-Loop-Helix Leucine Zipper (bHLHZip) transcription factor, which forms transcriptionally active dimers with another bHLHZip protein called Max [1,2]. Dimerization with Max endows Myc with sequence specific DNA binding ability, preferentially to sites containing the E-box sequence CACGTG. The transactivation properties of this complex are carried out by the N-terminal portion of Myc [3]. Myc is a multifunctional transcription factor able to regulate cell cycle, growth, metabolism, differentiation, apoptosis, transformation, genomic instability, and angiogenesis. Recently, transcription independent functions of Myc have been proposed particularly concerning the role of Myc in regulating pre-replication complexes assembly onto DNA replication origins [4]. While low Myc levels are necessary and sufficient for cellular viability and proliferation, pathological activation of this proto-oncogene has been linked to over-expression and gain of function mutations [5-7]. Pathological over-expression is frequently achieved by transcriptional up-regulation due to chromosomal translocation leading to promoter rearrangement [8-12], gene amplification [13,14] or by virus mediated insertional mutagenesis [15,16]. In prostate and breast cancers, a significant fraction of tumors demonstrate amplification of an otherwise unrearranged c-MYC locus (Pietilainen et al., 1995; Bubendorf et al., 1999; Sato et al., 1999; Naidu et al., 2002). In contrast, the Myc mRNA and protein over-expression that is observed in 70-80% of colon carcinomas (Smith and Goh, 1996) results from aberrant transcriptional control of the MYC locus involving mutations in APC-b-catenin-TCF-4 pathway members (Barker et al., 2000). Similarly, in Human acute T-cell lymphoblastic leukemias and lymphomas (T-ALL) gain-of-function mutants of Notch1 ensure robust transcriptional activation of MYC [17]. Besides, during tumor progression the Myc protein is often stabilized, either because it acquires specific point mutations [18] or because Myc turnover is regulated by oncogenic pathways such as RAS [19] or AKT [20].

### Myc activation elicits cell intrinsic tumor suppressive mechanisms

#### 1. The ARF/MDM2/p53 pathway

**Figure 1:**
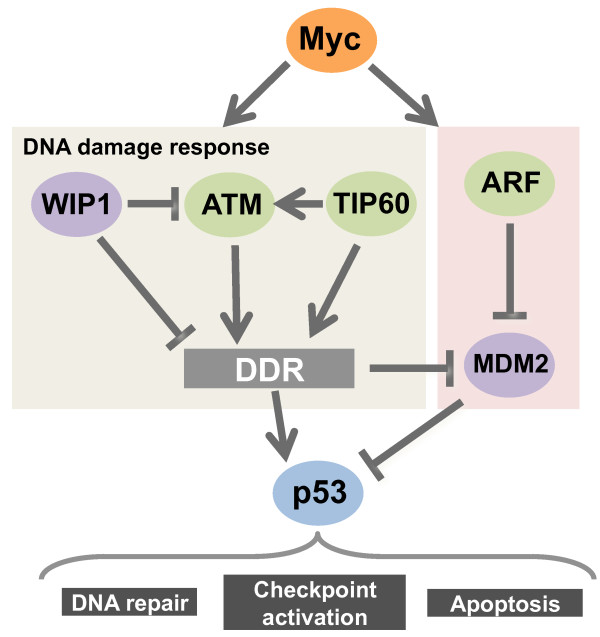
**Myc induced tumor suppressive pathways**. Outline of the p53 dependent pathways involved in Myc induced tumor suppression.

A direct consequence of Myc over-expression is a hyper-proliferative response, which is generally counter-balanced by the activation of intrinsic tumor suppressive mechanisms that effectively restrain clonal expansion of pre-cancerous cells. These mechanisms often arise as intracellular responses to stress situations directly induced by Myc. The best characterized arm of Myc-induced tumor suppression relies on the ARF/MDM2/p53 pathway, which results in the activation of a p53 dependent apoptotic response [21-23] (Figure 1). This pathway is controlled by ARF, a nucleolar protein, encoded by the INK4a/ARF locus, that is able to bind MDM2, a ubiquitin ligase that in turns ubiquitylates p53 and dooms it for proteasomal degradation. Thus this pathway is epistatically regulated by ARF levels: in normal conditions ARF is undetectable, while upon Myc-induced oncogenic stress its locus is transcriptionally activated, resulting in p53 stabilization and activation. Mouse model have been instrumental to the genetic dissection of the ARF/MDM2/p53 pathway and the characterization of the p53 effector functions required for tumor suppression, which, especially in hematopoietic malignancies rely largely, but not exclusively [24], on p53 dependent apoptosis [25,26].

#### 2. The myc-induced DNA damage response

The ARF/MDM2/p53 pathway is not the only tumor suppressive tool available to cell for restraining the oncogenic activities of Myc. More recently, the DNA damage response (DDR) has also been shown to act as an ARF-independent hurdle that limits aberrant cell division in early tumorigenesis [27-29]. Although it is still not completely clear what type of physical alterations are induced at the DNA level, several observations have led to surmise that at least two types of DNA damage can be associated with Myc overexpression. First, the production of reactive oxygen species (ROS), which have been shown to increase in experimental conditions where Myc is deregulated, can increase oxidative damage [30]. Indeed, the accumulation of ROS-associated oxidative damage coincided with transient MYC activation in human fibroblasts cultured in vitro in low serum (0.05%) and/or ambient oxygen tension [31]. To this end, it is worth mentioning that while anti-oxidants can reduce the Myc-induced DDR, their anti-tumoral activity has been mainly ascribed to their ability to affect the HIF1α pathway [32]. Another possible source of Myc-induced DNA damage is replication stress, a term used to define the generation of aberrant DNA replication intermediates which lead to the accumulation of DDR markers at sites of active DNA replication. The Myc-induced DDR accounts for the genomic and chromosomal instability frequently associated with Myc hyper-activation. Indeed, a number of chromosomal abnormalities including translocations, dicentric chromosomes and tetraploidy have been observed upon Myc overexpression [33]. Recent work has allowed the charting of the relevant pathways regulating Myc-induced DDR, leading to the identification of ATM [27,28,34], WIP1 [35] and TIP60 [29] as mediators of this response (Figure 1). ATM encodes a phosphatidylinositol-3-kinase-like protein kinase which is the apical kinase responsible for the activation of DDR following DNA double strand breaks (DSBs) accumulation. It phosphorylates and activates numerous substrates upon DNA damage, such as the diffusible kinase CHK2, which is required for proper cellular amplification of the DSBs induced DDR, and the p53 protein, the key effector of the DDR. Once phosphorylated, p53 escapes the deadly embrace of MDM2, ensuring a prolonged G2 arrest, the induction of DNA repair and, depending on the extent of DNA damage and on the cell type, stimulating either apoptosis or senescence [36]. Circumstantial evidence suggested a link between Myc and ATM, since ATM^-/- ^thymic lymphomas developing in mice ATM^-/- ^are frequently characterized by extra copies of chromosome 15, where the c-myc gene maps [37]. Also, oncogenic Myc activation has been associated with ATM inactivation in different human tumors, including B-cell lymphomas [38]. More direct evidence comes from a number of genetics studies in mouse models of Myc-induced tumorigenesis, such as the Eμ-myc transgenic mouse, which is predisposed to develop B-cell tumors with high penetrance [39]. In this genetic background, both DDR and apoptosis induced by Myc were reduced upon ATM loss, while tumorigenesis was markedly accelerated. Of relevance, ATM loss did not abolish the strong selective pressure to inactivate p53 or ARF, highlighting that ATM controls p53 independently from ARF [27,34]. The role of ATM in regulating Myc-induced tumor suppressive DDR, was also confirmed in a mouse model of skin cancer suggesting that the relevance of this pathway in suppressing Myc-induced tumors suppression is not restricted to hematological malignancies, but extends to solid tumors [28]. These results were extended by the genetic analysis of mice lacking WIP1 a protein phosphatase that negatively regulates ATM, with loss of WIP1 protecting against Eμ-Myc induced lymphomas [35]. WIP1 loss, not only abolished the genetic pressure for ARF loss of heterozygosity in tumors that developed in Eμ-myc;ARF^-/+^;WIP1^-/- ^mice, but also prevented tumor development in Eμ-myc;ARF^-/- ^mice. Yet, tumor suppressive responses were still completely dependent on p53. Thus the lack of WIP1 potentiates p53 dependent tumor suppression to a level sufficient to protect B-cells from Myc-induced lymphomagenesis, even in the absence of functional p19^ARF^. The enhanced tumor suppression conveyed by WIP1 loss was only partially bypassed by ATM loss, arguing for a more pleiotropic effect of WIP1 compared to ATM. Similar results were reported for the haploinsufficient tumor suppressor TIP60 [29], a histone acetyl transferase (HAT) that has been implicated in the regulation of the DDR via activation of ATM [40] and of DNA repair through the catalytic subunit of the NuA4 complex, a multifunctional complex with HAT and chromatin remodeling activities. The NuA4-Tip60 complex is recruited to DSBs, where it acetylates histones H2AX and H4, facilitating both turnover of H2AX and modifying chromatin architecture to allow DSB repair [41-43]. In the Eμ-myc transgenic mouse, TIP60 is a haploinsufficient tumor suppressor required for an efficient Myc-induced DDR. Loss of one TIP60 allele blunted ATM activation, p53 phosphorylation on Serine 18 and H2AX phosphorylation, resulting in a dramatic acceleration of tumorigenesis, while the activation of the ARF-p53 tumor suppressor pathway or the resulting apoptotic responses were unaffected. The occurrence of TIP60 mutations in human tumors and its parallel loss of expression in advanced stages of different cancer types suggested that mechanism of tumor suppression described in animal models are also relevant in human pathologies [29].

### Therapeutic implications of the evasion of DDR dependent tumor suppressive responses

The existence of tumor suppressive pathways evoked by the DNA damage response and their obligatory inactivation during tumor progression may have important therapeutic implications since some tumors may accumulate mutations in the DDR pathways in order to escape tumor suppression. These DDR defective tumors will be refractory to all the therapeutic regimens that require a functional DDR response, like for example many standard chemotherapy protocols [34]. On the other hand, tumors that are defective in some effector functions of the DDR may offer the opportunity for pharmacological targeting of specific branches of the DDR that are otherwise compensated in normal cells and tissues. This concept of synthetic lethality has been exploited for the rationale use of Poly (ADP-ribose) polymerase (PARP) inhibitors which generate replication dependent DNA double-strand breaks (DSBs) that are irreparable in BRCA1/2 defective cells thus effectively killing tumors [44]. Another example is represented by DDR checkpoint defective cells, which may be more sensitive to DNA repair inhibitors. In fact, CHK2 inactivation, which may be selected for during Myc-induced tumor progression may render cells more prone to DNA repair inhibition as shown in the case of Myc-induced tumors, where CHK2 loss shows a synergistic lethal response in combination with DNA repair inhibitors such as PARP inhibitors [45]. Also, mutations accumulated in DDR pathways during tumor development reduce the options available to a cell to respond to DNA damage thereby potentially exposing tumor cells to the inhibition of pathways that may be otherwise be compensated in normal cell. This concept is exemplified by the observation that ATM deficient cells are sensitized to the chemical and genetic inhibition of the ATR/CHK1 pathways, although in vivo validation of this concept in preclinical models is missing [46].

### The concept of oncogene induced replicative stress (OI-RS)

Oncogene induced replication stress has been recently recognized has a major component of the oncogenic stress response. Although still poorly characterized in terms of mechanism, replication stress is often associated with oncogene induced DDR (OI-DDR) [47]. Oncogenes such as Ras are capable of mounting strong proliferative responses that are frequently associated with elevated DDR activation. This association is particularly strong at the early stages of tumor progression in line with the hypothesis that OI-DDR represents a tumor suppressive response [47]. Several evidences lead to surmise that a strong component of the DDR activation relies on DNA damage triggered by replication stress [48,49]. First of all OI-DDR can be mimicked by over-expression of key regulators of the cell cycle machinery, such as cyclin E, whose activity peaks at the beginning of S-phase and in many systems contributes to S-phase progression, Cdc6 which is involved in replication origin licensing, the Cdc25 phosphatase, a positive regulator of all the cyclin/CDK complexes that are activated during S-phase, or E2F1. Second, in cellular models, over-expression of oncogenes leads to the accumulation of a DDR that depends on progression through S-phase: if DNA synthesis is pharmacologically blocked, then DDR activation is blunted. In addition, fragile sites, which represent genomics sites known to be prone to accumulate mutations during DNA replication, are hot spots for DNA mutations upon oncogene induced replication stress. Finally, the observation that H2AX phosphorylation and PCNA co-localize with single stranded DNA strongly suggest that a primary source of OI-DNA damage is generated at replication forks. All these evidences collectively suggest that OI-DDR is largely due to replicative stress.

### Myc ensures proliferative advantage by coping with replicative stress

**Figure 2:**
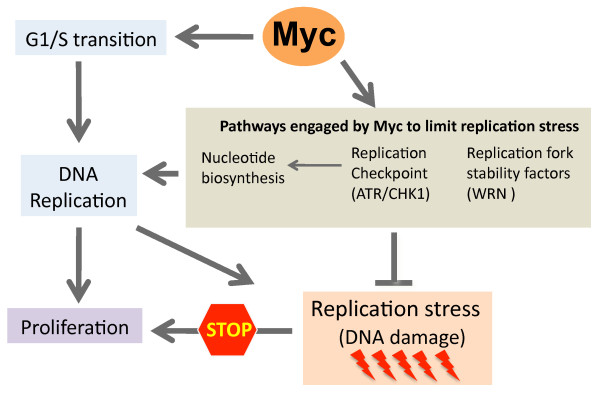
**How Myc deals with intrinsic replication stress**. Schematic representation of the pathways engaged by Myc to counteract intrinsic replicative stress responses that would limit clonal expansion of (pre)-cancerous cells.

As observed with many oncogenes, Myc over-expression brings about a certain level of replication stress documented by the generation of DDR markers in S-phase cells that co-localize with active replication forks. Yet, contrary to other oncogenes, Myc activation provides long-term proliferative advantage, thus suggesting that the anti-proliferative effects triggered by RS and DDR may be mitigated by Myc itself. In fact several evidence that will be presented below suggest that while unrestricted activation of Myc can generate replication stress, Myc also controls several pathways that actively restrain the extent of replication stress to allow proficient cellular proliferation (Figure 2).

**Figure 3:**
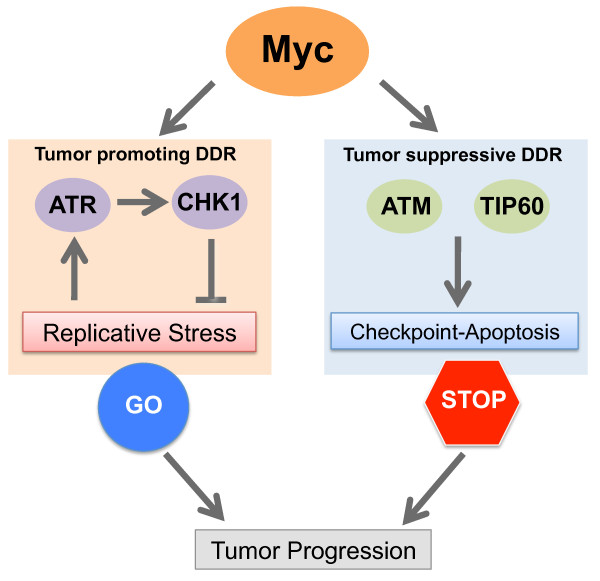
**Dual nature of Myc induced DDR**. Tumor promoting DDR: Myc engages the replication checkpoint pathway (ATR/CHK1) to allow robust cellular proliferation and avoid cytotoxic DNA damage accumulation. Tumor suppressive DDR: Myc induced DNA damage response triggers tumor suppressive pathways that act as a barrier to malignant transformation.

#### 1. Myc-induced DNA replication

The ability of Myc to promote cellular proliferation stems from its ability, as a transcription factor, to directly control the expression of a large number of cellular pathways implicated in S-phase progression [50]. In addition, rather unexpectedly, Myc itself can localize onto sites of active DNA replication suggesting a more direct role of Myc in controlling S-phase progression possibly by directly participating in licensing or assembly of (pre)-replicative complexes [4]. Alternatively, the pervasive Myc binding to the genome, especially when over-expressed, and its strong transcriptional activity may contribute to the activation of latent replication origin by making them more accessible to replication factors. Therefore we can envision Myc promoting S-phase either by transcriptionally regulating the cell cycle machinery, the nucleotide biosynthetic pathways and by directly affecting the number of active replication forks.

#### 2. How is myc-induced DDR activated during DNA replication?

We propose three alternative explanations, not necessarily mutually exclusive, as to how DDR is activated during S-phase in cells expressing elevated Myc levels. The first possibility is that the increased number of active origins and the increased global S-phase progression rate may raise the level of DNA damage physiologically accumulated during DNA synthesis. Alternative, the strong and pervasive Myc-induced transcriptional activity may generate the physical clashing of the RNA polymerase along with the DNA replication machinery thereby causing replication fork collapse and consequent activation of a DDR. Indeed, the co-localization of Myc, replication complexes and DDR factors onto DNA during S-phase, may be an indication that this situation happens in vivo. Lastly, DNA damage may be generated because of fork instability due to metabolic starvation, since despite Myc's ability to boost purine and pirimidine metabolism [51], this may not completely satisfy the high demand of nucleotides needed during hyper-proliferative S-phase.

#### 3. Myc overexpressing cells balance replication stress with S-phase progression

Despite evidences of a replication stress response in Myc over-expressing cells, a number of observations suggest that indeed elevated levels of Myc trigger also compensatory responses able to effectively reduce, but not completely eliminate, the consequences of replication stress. Here we provide some examples.

##### WRN helicase

The first example is represented by WRN, a gene encoding a RecQ DNA helicase, which is found mutated in Werner Syndrome, a disease characterized by cellular senescence, increased chromosomal instability characterized by frequent chromatid breaks, and accelerated aging [52]. The WRN helicase resolves topologically unfavorable DNA structures that form during S-phase, such as those arising at stalled replication forks, therefore representing a bona fide factor endowed with anti-replication stress activity [53]. MYC exacerbates this function of WRN, since its over-expression in WRN-/- fibroblasts leads to excessive accumulation of DNA damage at sites of newly replicated DNA [54]. This elevated DNA damage triggers the activation of the ATR-CHK1 pathway, resulting in a strong anti-proliferative, senescent response. Since WRN is a transcriptional target of MYC, up-regulation of the WRN gene represents a feed-forward mechanism to limit MYC-associated replication stress thus allowing continued cellular proliferation [55]. Grandori and coworkers also addressed the role of WRN in Myc associated cancer in xenografts experiments using a non-small cell lung carcinoma cell line expressing high levels of Myc (A549, NSCLC) where WRN was silenced by RNA knock-down. They also used a classic genetic approach breeding a cohort of Myc transgenics for Eμ-myc with mice carrying a germline mutation of WRN (Wrn^Δhel/Δhel^). In both cases tumor growth was significantly delayed, and as predicted, WRN loss of function in the context of robust Myc activation led to the elevation of a DNA damage response resulting in cellular senescence and tumor necrosis [56].

##### Nucleotide synthesis

Another trick Myc may pull off to counteract replication stress is increasing the rate of nucleotide synthesis. As already discussed, oncogenes are able enforce cell proliferation leading to replication perturbation, DNA damage accumulation and genome instability. This replication stress is at least in part due to the high rate of DNA synthesis which is not properly fuelled by the biosynthetic pathways that provide purine and pyrimidine nucleotides. In in-vitro experiments, an exogenous supply of nucleosides was shown to rescue replication stress, to decrease replication-induced DNA damage, and to reduce transformation of cells expressing viral or cellular oncogenes [57]. Shortage of intracellular nucleotides not only affects replication dynamics but can also modulate origin firing and licensing. Indeed, under chronic exposure to a low-nucleotide pool, cells have been shown to compensate for DNA replication stress by increasing origin density during G1. Recently, it was demonstrated that c-Myc is an important regulator of nucleotide biosynthesis and that its expression elevates nucleotide levels and cell proliferation [51,58]. This was experimentally validated, since replication stress induced by oncogenes such as cyclin E was significantly rescued by co-expression of c-Myc. Thus, the Myc-induced increase in nucleotide pool size can be regarded as an effective mechanism to rescue cells experiencing replication stress and to prevent DNA damage accumulation during S-phase.

##### The ATR/CHK1 pathway

Another pathway that is controlled by Myc and is involved in the replication stress response is the ATR/CHK1 pathway. This pathway is activated by Replication Protein A (RPA)-coated single stranded DNA (ssDNA), which can be produced during replication stress, DSBs resection, repair of DNA base adducts or DNA crosslinks [59,60]. The most common signal for ATR activation probably involves intrinsic replication stress in every S-phase and perhaps regulation of specific aspects of DNA replication such as origin firing or nucleotide production. As such, the ATR kinase and CHK1, are essential genes whose homozygous germline mutation compromises cellular viability and embryonic development [61-63]. The ATR gene encodes a large ATM-related protein kinase, which is recruited at sites of ssDNA along with ATRIP and is activated by the presence of accessory factors such as Claspin and TopBP1. Homozygous mutations affecting the splicing of the ATR mRNA have been identified in patients with the rare Seckel syndrome, a disease characterized by microcephaly and growth retardation. A mouse model recapitulating this mutation (ATR^s/s^) has confirmed its causality in determining the etiology of the disease, since ATR^s/s ^mice displayed accelerated aging, microcephaly, growth retardation and pervasive replication stress during embryonic development [64]. The other key component of this pathway is CHK1, a soluble checkpoint kinase activated directly by ATR, which contributes to the transduction of the DDR signals to activate effector functions of the pathway, such as the S and the G2 checkpoint. Other essential properties of CHK1 reside in its ability to regulate replication fork progression, regulation of nucleotide synthesis and anti-apoptotic activity [59,60]. Recent evidences have shown that the ATR/CHK1 pathway has a gatekeeping function essential to restrain replication stress in cells with activated oncogenes. In fact elevated levels of Myc sensitize a variety of cell types to chemical inhibition of either CHK1 or ATR, by exacerbating a strong DDR that triggers a potent apoptotic response [65-70].

### Exploiting replication stress to design novel anti-cancer therapies

The observation that Myc restrains intrinsic replicative stress responses by activating specific pathways has relevant implications from a therapeutic standpoint and holds the promise of prospective clinical applications. Indeed emerging evidences from recent in vivo studies suggest a potential anticancer effect achieved by specific targeting of the ATR/CHK1 pathway [65-70] or the WRN helicase [56]. Genetic experiments in mouse models of Myc-induced tumorigenesis such as Eμ-myc crossed with the ATR^s/s ^hypomorphic mutant unveiled a profound role of ATR in preventing inherent replication stress. In particular, a homozygous ATR^s/s ^background resulted in complete protection against lymphomagenesis [65]. A corollary of this synthetic interaction was the observation that, in ATR^s/s ^animals, even non lymphoid tissues with mild elevation of Myc levels (due to the leakiness of the Eμ-myc transgene during development) showed a pervasive RS that resulted in accelerated systemic aging and reduced life span. Consistent with the above results, systemic administration of the CHK1 inhibitor UCN01 in mice bearing fully blown lymphomas triggered a potent DDR response, cell cycle arrest and widespread apoptosis. Continuous administration of UCN01, or other CHK1 inhibitors, was able to cause fast regression of established lymphomas, thus indicating the therapeutic efficacy of targeting the ATR/CHK1 pathway. Unfortunately, the low solubility of available ATR inhibitors has so far limited their assessment in preclinical mouse models. These observations were confirmed by Nilsson and coworkers in a different mouse model of Myc-induced lymphomas [66]. Also, a functional RNAi screen in neuroblastoma cell lines that over-express N-Myc has recently led to the identification of CHK1 as a potentially therapeutic target [70]. In this study, sensitivity to CHK1 inhibition or knock-down positively correlated with N-Myc protein levels. Also, while CHK1 inhibition had little effect in primary non-transformed cells, the same cells modified to express high levels of N-Myc were highly sensitive to the treatment. Thus, CHK1 inhibition strategies represent a promising therapeutic option for the treatment of Myc-associated tumors.

### Concluding remarks

We have here discussed two opposing functions of Myc-induced DDR, which are relevant during pathological activation of Myc. First, the DDR exerts a tumor suppressive response that involves ATM, TIP60 and WIP1, resulting in the activation of p53. This effect of the DDR thus relies on p53 in order to restrains tumor development. As such, this represents the classical concept of OI-DDR generally associated with oncogene activation. The second function of the Myc-induced DDR involves the ATR/CHK1 pathway and has a completely different biological consequence, since it is part of the proto-oncogenic program that is activated in Myc overexpressing cells. This tumor promoting DDR, activated to support rather than suppress tumor development, acts to keep in a latent state tumor suppressive responses that would be otherwise toxic to cancer cells. This is one example of a number of cellular programs that are required to dampen tumor suppressive responses: others are represented by the need for cancer cells to have high levels/activity of specific Cyclin/Cdk complexes [71-74]. This rewiring of (pre)-cancerous cells to suppress tumor suppressive responses makes them addicted to the activity of specific pathways that are otherwise redundant in normal cells, thus opening up new therapeutic opportunities.

## Abbreviations

MYC: c-MYC; DDR: DNA damage response; OI: oncogene induced; OI-DDR: oncogene induced DNA damage response; OI-RS: oncogene induced replication stress; DSBs: DNA double-strand breaks; ssDNA: single stranded DNA; PARP: Poly (ADP-ribose) polymerase.

## Competing interests

The authors declare that they have no competing interests.

## Authors' contributions

SC and BA wrote the manuscript together. Both authors read and approved the final manuscript.
